# Aconite in Ephidrosis

**Published:** 1855-11

**Authors:** 


Aconite in Ephidrosis.—M. Imbert-Gourbeyne relates an interesting case of idiopathic general sweating, which, after continuing a long period, was immediately relieved by a few spoonsful of syrup of aconite, which he gave from recollection of the great benefit Dupont stated he had derived from this drug in a similar case. In another instance he found the same remedy efficient in the sweating of phthisis, when given for a coincident neuralgia.—(*Gaz. Médicale.* Nos. 21 and 22.) *Ibid.*

